# Impact of Gentamicin - Collagen Sponge (Collatamp) on the incidence of sternal wound infection in high risk cardiac surgery patients

**DOI:** 10.1186/1749-8090-8-S1-O123

**Published:** 2013-09-11

**Authors:** F Popescu, M Rochon, S Raja

**Affiliations:** 1 Cardiac Surgery, Harefield Hospital, London, UK

## Background

The purpose of this audit is to review the impact of local administration of Gentamicin – Collagen Sponge (Collatamp) on the incidence of sternal wound infection in high risk cardiac surgery patients within our hospital.

## Methods

This is a retrospective audit, data being collected over a period of 32 months between January 2010 and August 2012. All individuals received routine intravenous antimicrobial prophylaxis. Postoperative wound-infection rates as well as routine outcomes were compared. Information for the study was obtained from the cardiac surgical Patients Analysis and Tracking System (PATS) database and from hospital records.

## Results

Out of 2238 patients that were audited Collatamp was used in 122 patients (5%), of which 5% developed post – operative sternal wound infection. The type of sternal wound infection in Collatamp cohort was 60% deep infections (29% in non-Collatamp cohort), 40% superficial infections (56% in non Collatamp) and no organ/space infections (15% in non-Collatamp cohort). The rate of sternal wound infection in Collatamp cohort was raised (5%) in comparison with non-Collatamp cohort (3%), although the length of stay in hospital was shorter in Collatamp patients. No gentamicin sensitivity was recorded within the Collatamp cohort.

## Conclusions

Collatamp is an useful adjunct to meticulous surgical technique and postoperative wound care. Due to small numbers it is not possible to prove that it provides overwhelming benefits. No gentamicin resistance developed in patients treated with Collatamp.

